# Influence of cyclic AMP on facial nerve regeneration in rats

**DOI:** 10.1016/S1808-8694(15)31376-8

**Published:** 2015-10-17

**Authors:** Andrei Borin, Ronaldo Nunes Toledo, Paulo Lee Ho, José Ricardo Gurgel Testa, Oswaldo Laércio Mendonça Cruz, Yotaka Fukuda

**Affiliations:** 1 Master’s degree in otorhinolaryngology; doctoral student; 2 Biologist and doctorate in biochemistry; researcher at the Centro de Biotecnologia, Instituto Butantan; 3 Doctorate in otorhinolaryngology, adjunct professor, otorhinolaryngology discipline, UNIFESP/EPM; 4 Livre-docente (habilitation) professor, affiliated professor, otorhinolaryngology discipline, UNIFESP/EPM; 5 Livre-docente (habilitation) professor, otorhinolaryngology discipline, UNIFESP/EPM. Otorhinolaryngology and Head & Neck Department, Universidade Federal de Sao Paulo

**Keywords:** cyclic amp, histology, facial nerve, rat, nerve regeneration

## Abstract

Promoting facial nerve regeneration is a significant challenge.

**Aim:**

To evaluate the possible neurotrophic influence of cyclic AMP on facial nerve regeneration of Wistar rats.

**Method:**

The right facial nerve of thirty-two animals were completely transected and immediately sutured, followed by exposure or not to topical cyclic AMP. Behavioral and histometric analyses were done at 14 and 28 days.

**Results:**

Statistical differences (p<0.05) were found in the behavioral and histometric analyses on the 14th day, suggesting an early regenerative response of the facial nerve to cAMP exposure.

**Conclusion:**

This study demonstrates a possible neurotrophic effect of cAMP on facial nerve regeneration in rats.

## INTRODUCTION

In the well-known human anatomy book by Gardner/Gray/O’Rahilly, facial mimicry is described as being done by 22 muscle groups derived from the second branchial arch.^1^ These muscles are innervated by the motor division of the 7th (facial) cranial nerve. Injury to this nerve results in multiple dysfunctions that have cosmetic, functional and psychological impacts on such patients.^2^, ^3^, ^4^

The first attempts to optimize the regeneration of sutured nerves, using topical sulphonamide in the injury site, were undertaken in the Second World War.^5^ In 1951, Levi-Montalcini and Hamburger^6^ noticed that some tumors induced marked peripheral nerve growth, and suggested that these tumors probably produced endogenous substances that yielded this effect. Since then, controlled nerve injury has been used in experiments to study the effect of various physical, chemical and biological factors on nerve regeneration. In 1994, Heifti defined neurotrophic factors as those proteins that were able to regulate nerve cell survival and differentiation, nervous system development and integrity, and neuronal plasticity.^7^ This definition is currently being extended, since not only proteins have these actions, and since these factors have other properties, such as stimulating nerve growth and branching, inducing neuronal anabolism and modulating neurotransmission and electrical conductivity.^8^

Adenosine cyclic monophosphate (cAMP), which consists of adenylate cyclase and is degraded by phosphodiesterase, may be found in all eukaryotic cells;^9^ it is currently considered the main intracellular messenger molecule for a variety of cell stimuli.^9^, ^10^ Since 1955, research on the effect of glucagon on liver cells was started,^11^ cAMP has been held responsible for diverse events, such as controlling heart tone and inotropism,^12^ ovarian cell maturation and adrenal cell endocrine secretion.^9^ This molecule has also been associated with tumor cell resistance or susceptibility to chemotherapy,^13^, ^14^, ^15^ suggesting promising possibilities in cancer therapy. The herb Coleus forskohlii is used in traditional Hindu culture for treating insomnia, seizures and heart conditions; it contains the alkaloid forskolin, which raises the level of adenylate cyclase.^16^

A number of studies have clarified the role of camp in the neurological system. Clinically, it is involved in tumor growth, such as in astrocytomas, ependymomas and pituitary adenomas,^17^ and in neuronal protein expression in Alzheimer’s disease^18^ patients, among other effects. Results of cell culture studies showing that cAMP affects the survival and neuronal differentiation of PC 12 cells^19^, ^20^, ^21^, ^22^ and myelin production by Schwann cells^23^, ^24^ suggests that this nucleotide appears to have a neurotrophic role. Interneuronal synapse production^25^ and glial cell activity levels^26^ appear to be additional effects of cAMP. In animals, it seems to be associated with neuronal resistance to hypoxia in turtles during prolonged dives^27^ and with the extreme regenerative abilities of the spinal cord in fish.^28^ Sciatic nerve recovery in rats was tested in the 1970s, with conflicting results.^29^, ^30^ New promising results in sciatic nerve recovery were published in the 1980s.^31^, ^32^ However, we found no published paper on the role of cAMP in mammal facial nerve regeneration; this then became our theme for study.

## METHOD

The Research Ethics Committee at our institution approved this research project (number 1708/05). Male Wistar rats weighing between 200 and 240 g, kept in adequate cages in a climatized room under a 12-hour day-night cycle and with free access to water and food, were used in this study. There were four animal experiment groups, as follows: “cAMP 14D”: complete transection of the right facial nerve trunk, followed by prompt suturing and exposure of the injury site to cAMP and sacrifice 14 days later; “CTRL 14D”: the same injury and suture with exposure to Ringer’s lactate (RL) and sacrifice 14 days later; “cAMP 28D”: the same injury and suture with exposure to cAMP and sacrifice 28 days later; and “CTRL 28D”: the same injury and suture with exposure to RL and sacrifice 28 days later. The initial procedure for right facial nerve trunk transection and suturing was done in accordance with the established description for an experimental model by our group.^33^ Osmotic pumps (Alzet® Mini-osmotic Pump model 2002) coupled to a polyethylene catheter flowing at 0.5μl/hour for 14 days was used for releasing cAMP into the neural suture site. These pumps were placed subcutaneously in the interscapular region during the first procedure; the catheter tip was fixed in place with prolene 7-0 sutures at about 2mm from the neural suture. A solution containing dibutyril-cAMP 1mM salt manufactured by Sigma® (“N6,2´-O-dibutiryladenosine 3´:5´-cyclic monophosphate”) diluted in RL was used in groups exposed to cAMP. Pumps contained only RL in the other groups.

Animals underwent behavioral observation of facial mimicry in alternate days after surgery until 14 or 28 days, depending on the group, to observe vibrissal movement and closure of the palpebral rima. Scores for these observations have been described by Borin et al.;^33^ each item is scored 0 to 5 points, noting the progression of movement recovery.

The animals were sacrificed after 14 or 28 days, and the right facial nerve was dissected to locate the suture line and to remove a distal fragment for histological analysis. The contralateral facial nerve trunk (left) was also removed from all rats and sent for histological analysis. Biopsy material for histology and the digitized microscopy images of the facial nerve were processed in the Electron Microscopy Center (Centro de Microscopia Eletrônica - CEME) at our institution, according to a previously described protocol.^33^ Pictures were displayed sequentially according to the time between injury and sacrifice and exposure or not to cAMP for qualitative histological analysis; the presence of myelinated fibers, their relative diameter and the uniformity of their distribution within the nerve were noted. Myelinated fibers in right and left facial nerve trunks in each rat were counted^33^ and the right/left ratio was obtained for the quantitative histological analysis (histometry). The area studied in each nerve was 6399.679μm^2^.

The statistical analysis of data was based on two associated events: intervention (drug - administration or not of cAMP) and time (14 or 28 days). Data from each group were analyzed as follows:

1) clinical score for vibrissal movement;
2) clinical score for closure of palpebral rima;
3) fiber count in the left facial nerve;
4) fiber count in the right facial nerve;
5) right-left fiber count ratio. The analysis of variance (ANOVA) test was used for the comparative analysis to detect possible differences among group means, followed by a POST-HOC test. The significance level was 5%.

## RESULTS

The first procedure was done in 32 animals, eight in each experimental group. One week after surgery we noticed a soft tumor that had formed around the osmotic pump in one of the animals in the cAMP group; this animal was sacrificed, and the finding was an encapsulated inflammatory process containing pus. We discarded this animal from the sample and operated another rat for the 8-animal group. Thus, 33 animals were used in this study.

After performing euthanasia we had a subjective impression that there was more fibrosis in the dissection of the right facial nerve trunk in the animals belonging to the groups cAMP 14D and cAMP 28D. Furthermore, their nerves were apparently thicker (increased diameter); these subjective impressions were written in the observation sheets. We found no difficulties in locating the site of the epineural suture done in the first procedure; a distal segment was removed in all animals.

### Behavioral Observation

**Graph 1:**
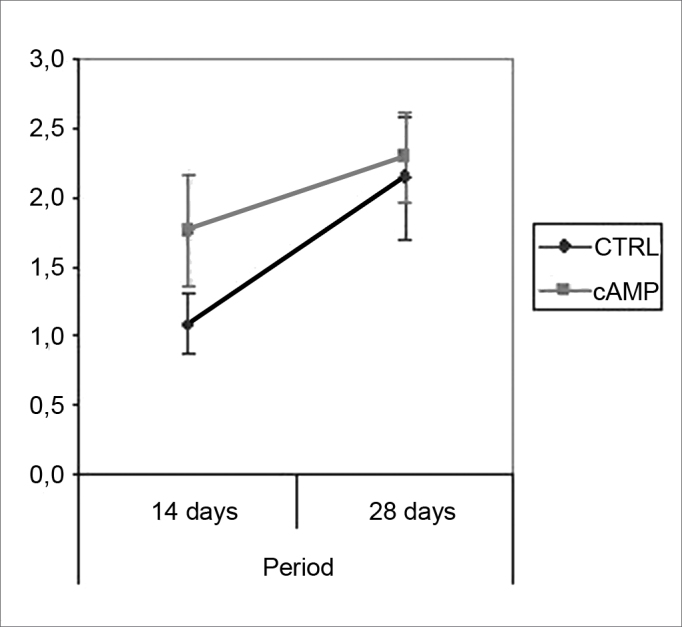
Clinical scores for palpebral rima closure. Confidence interval for the mean: mean ± 1.96 * standard deviation / *√* (n-1)

**Graph 2:**
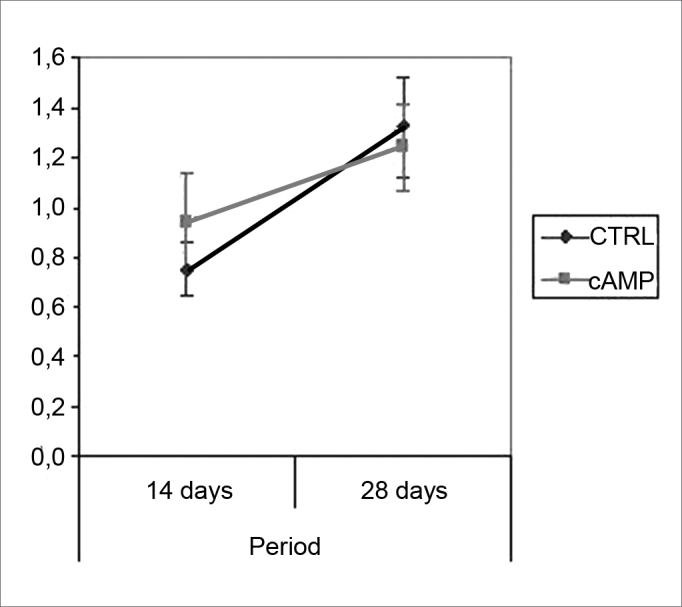
Clinical scores for vibrissal movement. Confidence interval for the mean: mean ± 1.96 * standard deviation / *√* (n-1)

We found improved vibrissal movement and closure of the palpebral rima in all groups. The mean clinical scores (and standard deviations) are presented in Table 1;,Graphs 1 and 2 show the confidence interval of the analysis. We also found that regardless of exposure to cAMP, animals sacrificed in the 28th day had better results compared to those sacrificed in the 14th day. The cAMP 14D group had superior results compared to the CTRL 14D group in closure of the palpebral rima, but not in vibrissal movement.

**Table 1:** Clinical score (mean (standard deviation)).

	cAMP 14D	CTRL 14D	cAMP 28D	CTRL 28D
Vibrissal movement	0,946	0,750	1,241	1,321
	(0,264)	(0,148)	(0,235)	(0,273)
Closure of palpebral rima	1,768	1,089	2,295	2,143
	(0,540)	(0,295)	(0,436)	(0,589)

### Qualitative Histological Analysis

**Figure 1:**
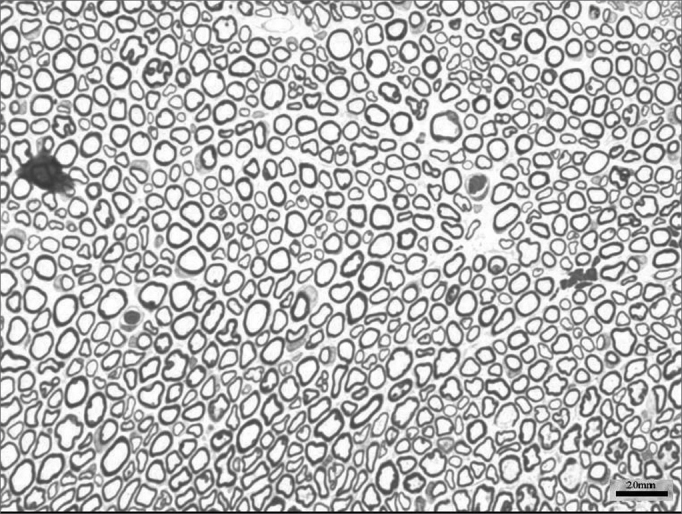
Typical aspect of the left facial nerve (UNINJURED) in all groups.

We obtained 64 images of the right and left facial nerves of each of the eight animals in each group. Images of the left facial nerve (not injured) in all animals showed no differences; their myelinated fibers were evenly distributed throughout all images, as shown in Fig. 1.

**Figure 2:**
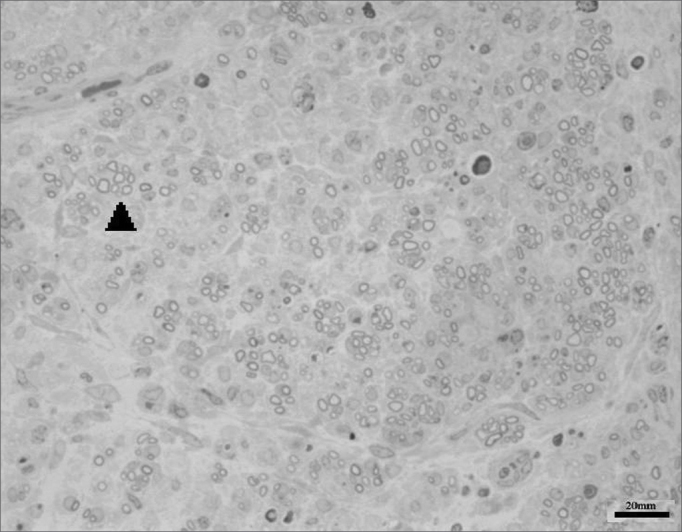
Typical aspect of the right facial nerve in the AMPc 14D group. Arrowhead shows clusters of small myelinated fibers

**Figure 3:**
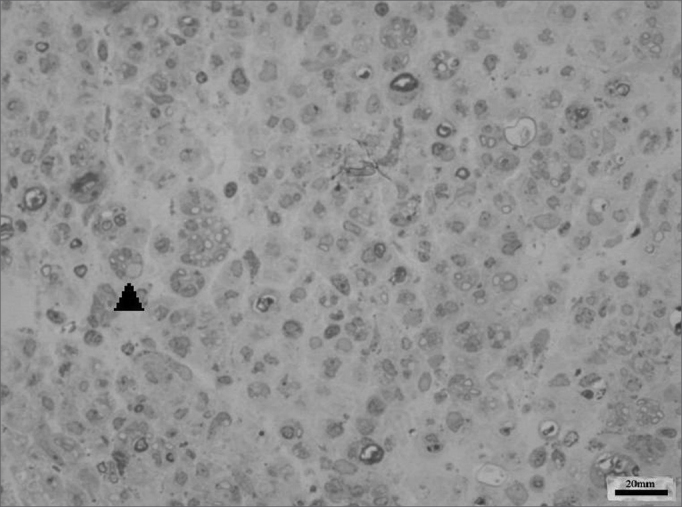
Typical aspect of the right facial nerve in the CTRL 14D group. Arrowhead shows clusters of small myelinated fibers

Images of the right facial nerves in groups CTRL 14D and cAMP 14D (injured nerves) showed small myelinated fibers with a lower diameter compared to the corresponding contralateral images; these fibers were often grouped rather than evenly distributed throughout the field. These findings appeared somewhat more evident in some animals compared to others; they were also more evident in group cAMP 14D compared to the group CTRL 14D. Figs. 2 and 3 show these results.

**Figure 4:**
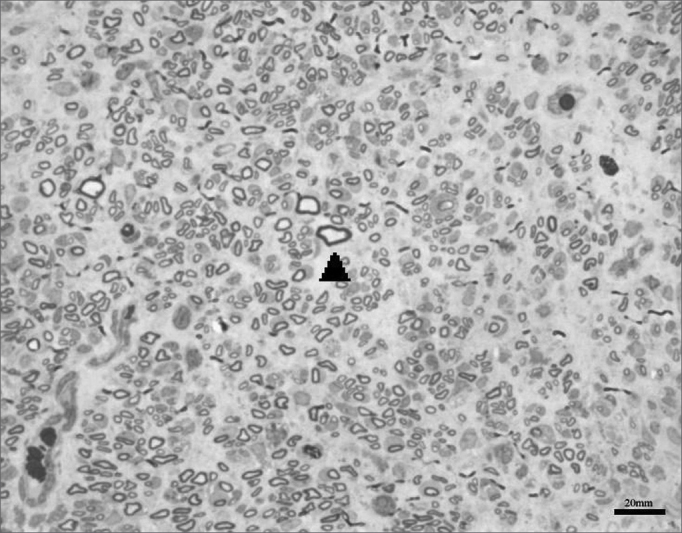
Typical aspect of the right facial nerve in the AMPc 28D group. Arrowhead shows larger diameter myelinated fiber

**Figure 5:**
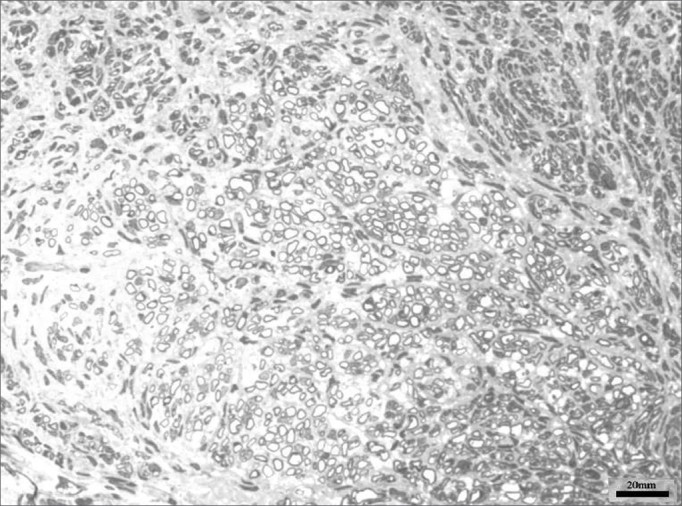
Typical aspect of the right facial nerve in the CTRL 28D group

The right facial nerves in groups cAMP 28D and CTRL 28D had more of these small myelinated ungrouped fibers, although there were variations between animals. There were also fibers of a larger diameter that were more similar to the left facial nerve. Figs. 4 and 5 show each of these groups.

### Quantitative Histological Analysis (Histometry)

**Graph 3:**
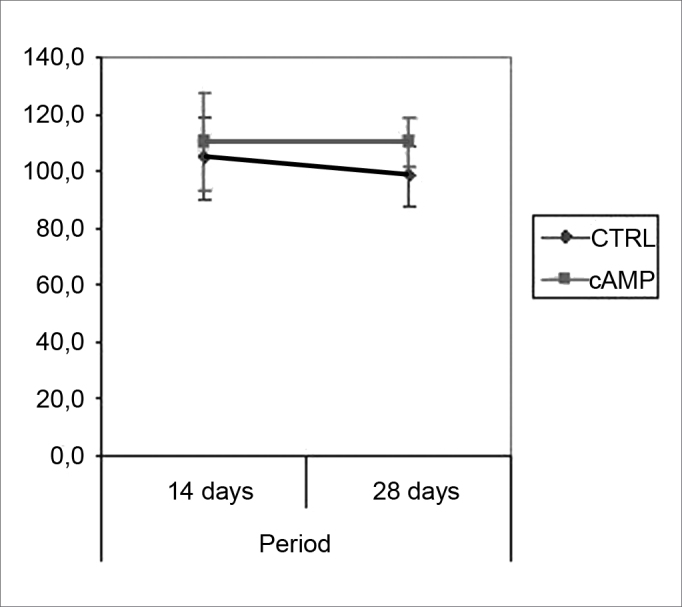
Fiber count in the left facial nerve. Confidence interval for the mean: mean ± 1.96 * standard deviation / *√* (n-1)

Fiber count means (and standard deviations) in left and right facial nerves and the right/left ratio are shown on Table 2; a comparative analysis is shown on Graphs 3 and 5. The left facial nerve (not injured) count did not vary among the four groups. The right facial nerve (injured) count, and the right/left ratio was higher on the 28th day compared to the 14th day, regardless of the presence of cAMP, suggesting that regeneration occurred along those periods. After 14 days the drug concentration was higher in the cAMP 14D group (32.6 ±24.2) compared to the group CTRL 14D (9.4 ±11.5); the right/left ratio was 31% × 9.7%. After 28 days, the drug concentration was higher in the CTRL 28D group (123.2 ±16.8) compared to the cAMP 28D group (92.1 ±13.0).

**Graph 4:**
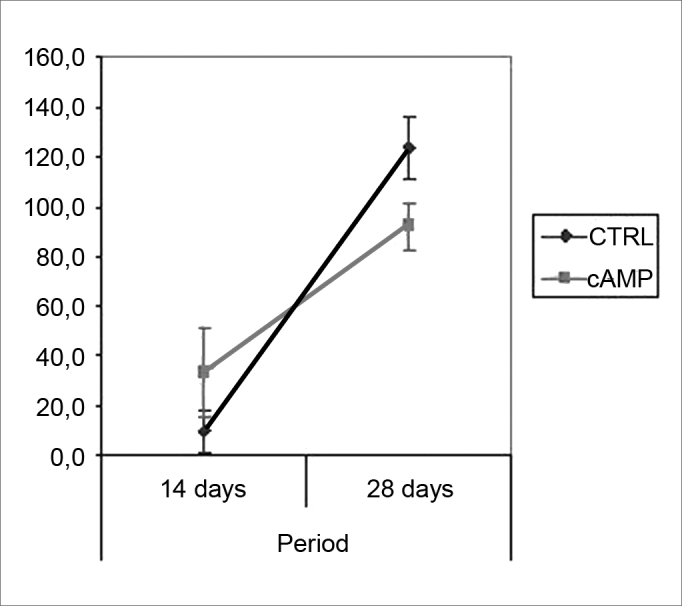
Fiber count in the right facial nerve. Confidence interval for the mean: mean ± 1.96 * standard deviation / *√* (n-1)

**Table 2:** Myelinated fiber count (mean (standard deviation)).

	cAMP 14D	CTRL 14D	cAMP 28D	CTRL 28D
Left facial nerve	110,375	104,750	110,125	98,625
	(23,360)	(19,667)	(11,457)	(14,232)
Right facial nerve	32,625	9,375	92,125	123,250
	(24,160)	(11,488)	(13,021)	(16,816)
Right/left ratio (%)	30,603	9,695	84,590	127,531
	(24,090)	(11,913)	(15,472)	(26,981)

**Graph 5:**
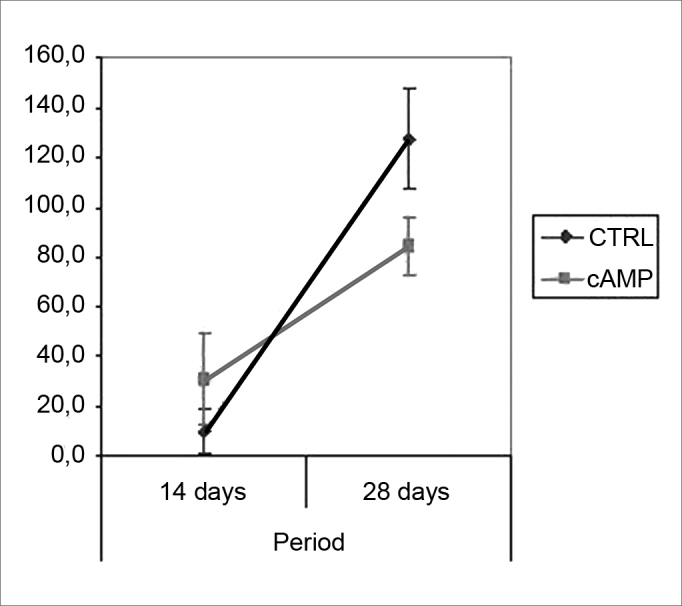
Right/left ratio - fiber count. Confidence interval for the mean: mean ± 1.96 * standard deviation / *√* (n-1)

## DISCUSSION

In 1972, Appenzeller and Palmer^34^ found that cAMP levels were increased in regenerating nerves. Since then various authors have raised the possibility that cAMP might have a neurotropic role; this has been further tested experimentally “in vitro” (cell cultures).^23^, ^24^, ^25^ Studies on the effect of cAMP on sciatic nerve regeneration in rodents have yielded variable results, with nerve stimulation in some^29^, ^31^ and absence of stimulation in others.^30^, ^35^ Much of this apparent conflict may be explained by the methods. The model we used in this paper (sciatic nerve crush injury) does not, in our view, cause homogeneous injury; crushing time ranged from 2 to 30 seconds, and the pressure was not measured. The time elapsed before analysis of results also varied in these papers; positive results were found in studies by Pichero et al.^29^ and Gershenbaum and Roisen^31^ after the 12th day, while studies by McQuarrie et al.^30^ and Black and Lasek^35^ were ended earlier (9 and 8 days) and therefore revealed no neurotrophic effects.

In 1984, Kilmer and Carlsen^36^ applied topical forskolin, which raises the levels of adenylate cyclase, thus increasing endogenous cAMP, achieving positive results in sciatic nerve regeneration in frogs. In 1987, these authors suggested delivering cAMP in pellets into the nerve injury site of hamster sciatic nerves, and found promising results.^32^ Studies on the role of exogenous cAMP should assure that this substance truly penetrates de cell for its intracellular action, as there are no extracellular receptors for this substance.^37^ The analogues dibutyril-cAMP and 8-bromo-cAMP pass through the plasmatic membrane and are less susceptible to phosphodiesterase action; their effect, therefore, is more evident than that of natural cAMP.^13^, ^38^ We believe the topical route is preferable for growth factors; if eventually it becomes used in humans, the dose and side effects would be lower compared to systemic use.^4^ Additionally, neurotrophic factors affect other cell groups, which in theory might stimulate tumor growth elsewhere when used systemically; a similar conundrum is estrogen hormone replacement therapy in patients with a family history of breast cancer. Another advantage of topical use is that the contralateral nerve may be used as a control for the injured nerve in experimental studies.^4^, ^39^

In our method we decided to transect completely and suture the nerve since this is a more reproducible injury; it is always classified as grade V (Suderland);^39^, ^40^ it also best reproduces the surgical correction of nerve injury in human beings.^41^ We also chose to deliver dibutyril-cAMP topically for the reasons given above. We also chose to extend our investigation to 28 days for increased reliability and to add behavioral and histological evaluations for increased objectivity. A deeper debate about our method may be found in Borin et al.^33^ We understand that our type of suture is more an approximation of neural stump rather than a classical microsurgical suture, since it was technically impossible to place more than one suture stitch in the facial nerve of rats.

Our behavioral analysis showed recovery of function accelerated by cAMP when tested by closure of the palpebral rima, but not by vibrissal movement, in the 14th day; this effect was not seen in animals tested after the 28th day. This finding has been reported by other authors, who have stated that the earliest sign of recovery of the facial nerve in rodents is partial recovery of the blink reflex.^42^ Komura et al. (1999)^4^ investigated the regenerating effect of the brain-derived neurotropic factor (BDNF) in the rat facial nerve, and found similar results, with earlier clinical recovery within the first 14 days and loss of accelerated recovery after 23 days. It appears to us that cAMP may stimulate early recovery, but not the end functional results; we discuss this further when commenting our histological findings. It is clear that the analysis of facial mimicry in rats is limited in that it does not allow any perception of nuances in expression compared to the scales used in human beings. Thus we lose the ability to detect subtle differences in motor recovery of the rat face at more advanced recovery stages.

We might find it curious that the animals in this study had facial movements so soon. Our methodological pilot study^33^ had already detected that about 35% of facial function returned within three weeks and 60% in five weeks. Komura et al.^4^ noted early recovery (14 days) following complete transection and suture of facial nerves in rats; Gershbaum and Roisen^31^ were surprised with the fact that the rat sciatic nerve showed clinical activity within two to three weeks of injury. This could be due to different crossed innervation compared to human anatomy, which is reflected in the activity of the contralateral facial nerve, as some authors have suggested,^43^, ^44^, ^45^ and/or a higher regeneration rate in rat compared to humans, as other authors have suggested.^31^

Fiber count of the left facial nerve (not injured) did not vary significantly among groups in this study. This finding confirms not only the findings that describe the lack of variability of this inter-species criterion, even when weight and age are different,46 but also the possibility of using samples of the non-injured size as controls for the injured side in neural regeneration experiments.^39^, ^47^, ^48^ Data showed a significantly increased myelinated fiber count in the right facial nerve (injured side) on the 14th day in the cAMP group compared to controls, a finding that was confirmed by the right/left ratio analysis. Apparently, cAMP stimulated early neural regeneration, accelerating axonal sprouting from the proximal stump. More fibers were found and our qualitative histological findings showed fine fibers grouped in blocks, which may mean a cross-section of neural sprouting, composed of the small immature fibers described by other authors.^30^, ^49^, ^50^, ^51^ Using electron microscopy, Gershenbaum and Roisen^31^ showed that cAMP accelerated the onset of Wallerian degeneration on the 3rd day after injury in the rat sciatic nerve model; there were also more fibers on the 10th day in the group exposed to cAMP, compared to the group exposed to saline.

Various authors have suggested numerous mechanisms by which topical and systemic cAMP might stimulate neural regeneration, although many doubts remain.^31^, ^32^ Genic expression effects^14^, ^27^ and cytoskelelal changes^36^, ^38^ in a variety of cell groups involved in neural regeneration have already been demonstrated. It is thought that cAMP may stimulate neuronal populations and also support structures, such as microglia, macrophages and Schwann cells.^23^, ^24^, ^25^, ^26^ It would thus “clean up” the injury site, removing cell remains, accelerating Wallerian degeneration, myelination and the neuronal growth sprout by acting on the neuron cell nucleus and/or the injury site.^31^, ^32^, ^34^, ^36^ Our study did not include the investigation of the mechanism of action of cAMP, so we accept these hypotheses.

On the 28th day we found an inversion in the previous hystometric finding, in which the fiber count in the control group and in contralateral nerves (right/left ratio = 127%) was higher than that in the group exposed to cAMP. There are a few possibilities for explaining this apparently conflicting result. In our experiment, the osmotic pump delivered cAMP only during the first 14 days; the remaining two weeks were nucleotide-free. This could suggest that nucleotide action might take place only if it is present, and that initially hyperstimulated fibers would be eventually lost. Furthermore, other authors have suggested that sprouting is stimulated by local factors such as cAMP, while neurite outgrowth depends on metabolic changes in the neuronal nuclear body, and therefore topical delivery would lose its effect with time.^32^ Concerning the higher fiber count in the right side compared to the left side in the CTRL 28D group, McQuarrie et al.^30^ found that after unilateral sciatic nerve injury, the number of fibers in the injured site was 40% higher compared to the uninjured side; these authors suggested that multiple sprouting from a single axon in the proximal stump might cause these findings. Byers et al.^52^ have described similar findings in a study of rat facial nerve regeneration.

In cell cultures, neurotrophic factors may induce cell differentiation and phenotypical changes, followed by decreased cell numbers. For instance, in cells originating from neuroblastomas, cAMP increases differentiation by stimulating new dendrites, at the same time decreasing the total number of cells.^53^ In our studies we probably witnessed early axonal sprouting and a decreased total number of regenerating fibers as a secondary effect. If so, the final functional results would not be altered, as the behavioral analyses in groups cAMP 28D and CTRL 28D were similar.

A further explanation is that during regeneration, the many fibers present initially in sprouting are gradually selected as “winners,” their diameter and myelination is increased to strengthen conduction, and inefficient fibers are lost.^31^, ^41^, ^48^, ^49^, ^50^, ^51^ Komura et al. 1999^4^ analyzed neural regeneration by expression of GAP43 protein mRNA, which affects axonal outgrowth. These authors found more significant clinical recovery in the BDNF-exposed group in the 14th day, followed by increased GAP43 protein mRNA; in the 28th day, behavioral findings were similar between the control and BDNF groups, with increased GAP43 protein mRNA expression in the control group at this phase. They attributed these findings to the fact that the neurotrophic factor stimulated group is at a more “advanced” regeneration step in which GAP^43^ protein expression is already lower. Similarly, we might suggest that the regeneration process in the cAMP 28D group was in a more advanced phase, suggesting a stage beyond sprouting, while the CTRL 28D group probably was in an earlier phase in which fiber numbers, rather than efficiency, were increasing; functionally, both groups had similar behavioral results.

Unfortunately our paradigm is limited and does not allow us to reject or accept any of these hypotheses. Different methods, such as more prolonged exposure to cAMP, direct and/or systemic availability to the central nuclei of the facial nerve, histological analyses with other time periods (such as the 21st day and after the 28th day), and methods for quantifying myelination and fiber diameter, are suggested as continuation for this study.

## CONCLUSION

We consider having found behavioral and histometric evidence of a neurotrophic role for cAMP on facial nerve regeneration in rats undergoing full nerve transection and suture.
